# Professor J. K. Mitchell

**Published:** 1858-05

**Authors:** 


### NECROLOGICAL NOTICES.

DEATH OF PROFESSOR MITCHELL.—It is our painful duty to announce the death of Dr. John K. Mitchell, Professor of Medicine in Jefferson Medical College. It took place at his residence in this city, on the evening of the 4th instant, in consequence of an attack of pleuro-pneumonia of only one week's duration. His illness was very sudden, and the news of the fatal event cast a gloom over the whole community, as we doubt not it will over the whole profession of the United States. Several years ago, Dr. Mitchell had an apoplectic seizure, from which, although it was followed by partial paralysis that continued down to the time of his death, he yet sufficiently recovered to enable him to deliver, with his accustomed ability, his annual courses of lectures, with hardly any interruption. During the last winter, indeed, his health was so good that he was obliged to absent himself from his class only on a single occasion.

This is neither the time nor the place to present a biography of the lamented deceased. This melancholy duty has been already assigned, as will be perceived by the subjoined resolutions of the Faculty of Jefferson Medical College, to other hands, and we cannot doubt that it will be executed with becoming taste, judgment, and fidelity. We cannot, however, forbear adding a few remarks respecting the most prominent features of his career.

Dr. Mitchell was a native of Virginia, having been born at Shepherdstown, in that State, on the 12th of May, 1793. He was, consequently, at the time of his death, nearly sixty-five years of age. His father was a Scotch physician, who emigrated to this country toward the close of the last century, and died before his son, who was destined to shed so much lustre upon his name, had attained his ninth year. At the age of fourteen young Mitchell, left with a small patrimony, to the care of a guardian, was sent to the village of Ayr, in Scotland, to receive his academical education. After a sojourn there of several years, he was transferred to the University of Edinburgh, then in the zenith of its renown as a literary and scientific institution, and while there he had the good fortune to make the acquaintance of Jeffry, Wilson, and other men, who subsequently performed so brilliant a part in Scottish literature. Returning to his native country in 1816, he studied medicine under Dr. Kramer, of Virginia, and afterward under Professor Chapman of this city, attending at the same time a course of lectures in the University of Pennsylvania. His health, however, failing him, he was advised to make a voyage to China, and he repeated his visit to that country after he took his degree in 1820. Settling in

Philadelphia, he became a candidate for business, published a small volume of poems, and in 1822 was appointed physician to the Philadelphia Alms House, a position which he resigned six years after for the same office in the Pennsylvania Hospital. Prior to this period he was already engaged in teaching chemistry, a lectureship having been tendered him in the Medical Institute of this city, founded by his illustrious preceptor, Dr. Chapman, between whom and himself there always existed a warm personal friendship, which only ceased with the death of the former in 1853. It was here, while associated with such men as Jackson, Hodge, John Bell, and others of a similar character, that Mitchell performed his series of valuable experiments upon the "Penetration of Gases," a subject of great physiological interest, and which, until then, had hardly received any attention either in Europe or in this country. The results of these experiments were embodied in a paper which he published in the *American Journal of the Medical Sciences*, and which elicited at once the notice of the learned in both hemispheres.

In 1841, Dr. Mitchell was appointed Professor of Medicine in Jefferson Medical College, a position in which he shone to peculiar advantage, for it afforded him, for the first time in his professional life, an opportunity for the full display of his rich and varied talents as a public teacher. The school, hitherto depressed by adverse circumstances, rapidly rose into distinction, and to Mitchell and his associates belonged the credit of having lectured, at one time, to the largest medical classes that had ever been assembled anywhere in the world. His presence in the lecture-room was peculiarly pleasing and captivating; his sweet, melodious voice, his manly appearance, his agreeable manners, his apt illustrations, and his ready anecdotes, rendered him one of the most attractive of teachers; while, personally, he enjoyed universal popularity with his pupils.

Early in life, Dr. Mitchell was a copious contributor to the medical press of this city, especially to the *North American Medical and Surgical Journal*, whose pages bore frequent evidence of his erudition and scientific attainment. All his papers were characterized by grace of diction, accuracy of narration, and an easy flow of language, which served to impart to them a charm seldom seen in similar writings. We believe he never attempted the composition of any elaborate systematic work. About ten years ago he published a monograph on the "Cryptogamous Origin of Malarial Fever," which attracted much attention at the time, on account of its ingenious reasonings and argumentations. For many years he enjoyed a large and lucrative practice.

Dr. Mitchell possessed a mind eminently poetical and susceptible of the beautiful. Whatever was lovely in nature was sure to attract his attention, and become the subject of comment. His studies were not purely professional, although these claimed much of his consideration, but they embraced almost every topic in literature, the arts, and sciences, upon all of which he conversed agreeably and intelligently. Of an eminently social disposition, he was the charm alike of the social circle and of the festive board, at which he not unfrequently presided on public occasions. Courteous and affable in his manners, he was one of the most popular of men and physicians; an excellent husband, an affectionate, judicious father, a devoted friend, a virtuous citizen. He has left behind him a wife and six children, of whom Dr. S. Weir Mitchell bids fair to transmit his father's name and fame with undiminished lustre.

The following is a minute of the action taken by the Faculty of Jefferson Medical College, soon after the death of their late colleague, the Dean, Professor Dunglison, being appointed under the second resolution :—

"The Faculty of Jefferson Medical College, on the occasion of the decease of their distinguished colleague, Professor J. K. Mitchell, are desirous of recording their estimate of his rare qualities as a professor and an associate.

"Ever since the reorganization of the school, in the year 1841, Professor Mitchell took a zealous and active part in its management, and in the important instruction delivered therein; and it may be safely affirmed, that in his anxiety and well-directed efforts for the best interests of the Institution, and of the vast number of pupils who have flocked to its halls, he has been exceeded by no one.

"As an associate, his deportment to his brethren of the Faculty was ever courteous and conciliatory; and in the numerous acts which led to the unprecedented success and development of the school, he participated largely, energetically, and ably. The loss of one so admirably qualified for his responsible office as a professor, of an associate so endeared, is, indeed, most profoundly mourned by his colleagues.

"*Resolved*, That an obituary notice of the deceased be delivered before the class of next session by one of his colleagues.

"*Resolved*, That a copy of the above minute be communicated to the family of their late friend and colleague, with assurances of the heartfelt sympathy of the Faculty in the grievous affliction which has befallen them."

